# Child Allergic Symptoms and Mental Well-Being: The Role of Maternal Anxiety and Depression^☆^

**DOI:** 10.1016/j.jpeds.2014.05.023

**Published:** 2014-09

**Authors:** Alison Teyhan, Bruna Galobardes, John Henderson

**Affiliations:** School of Social and Community Medicine, University of Bristol, Bristol, United Kingdom

**Keywords:** ADHD, Attention deficit/hyperactivity disorder, ALSPAC, Avon Longitudinal Study of Parents and Children, SEP, Socioeconomic position, TDS, Total difficulties score

## Abstract

**Objective:**

To determine whether maternal mental health mediates the relationship between eczema or asthma symptoms and mental well-being in children.

**Study design:**

Analysis of 7250 children from the Avon Longitudinal Study of Parents and Children. Child mental well-being at 8 years was measured by the Strengths and Difficulties Questionnaire. Binary outcomes were high ‘internalizing’ (anxious/depressive) and ‘externalizing’ (oppositional/hyperactive) problems (high was >90th percentile). Child rash and wheeze categories were ‘none’; ‘early onset transient’ (infancy/preschool only); ‘persistent’ (infancy/preschool and at school age); and ‘late onset’ (school age only). Maternal anxiety and depression were reported during pregnancy and when child was 8 years old.

**Results:**

Persistent wheezing symptoms were associated with high externalizing (OR 1.74, 95% CI, 1.41-2.15) and internalizing (1.67, 1.35-2.06) problems compared with never wheeze. Maternal anxiety and depression, and disrupted child sleep, attenuated these associations. Persistent rash (externalizing: 1.74, 1.40-2.15; internalizing: 1.42, 1.16-1.74) and late onset rash (externalizing: 1.62, 1.17-2.25; internalizing: 1.46, 1.07-1.99) symptoms were associated with poorer mental well-being compared with no rash at any age. Maternal anxiety and depression, particularly when child was aged 8 years rather than during pregnancy, accounted for the association with internalizing symptoms and partly for externalizing symptoms. Sleep disruption did not mediate the association.

**Conclusions:**

Maternal anxiety and depression may mediate the association between child rash and wheeze and child mental well-being.

Children with eczema and asthma have been reported to have poorer mental well-being than healthy children; childhood asthma has been associated with anxiety, depression, emotional and behavioral problems, and treatment for a mental health problem,^1^, ^2^, ^3^, ^4^, ^5^, ^6^, ^7^, ^8^, ^9^ and childhood eczema with increased emotional problems and a higher risk of attention deficit/hyperactivity disorder (ADHD).^10^, ^11^, ^12^, ^13^, ^14^

Childhood eczema and asthma are also associated with maternal anxiety and depression; mothers who are anxious or depressed during pregnancy, the postpartum period, and beyond are at increased risk of having a child with asthma or wheezing.^15^, ^16^, ^17^, ^18^ There is also evidence of reverse causation; caring for a child with eczema or asthma is associated with higher levels of anxiety, depression, sleep deprivation, and reduced quality of life for the child's parents and family.^19^, ^20^, ^21^, ^22^, ^23^, ^24^

Therefore, as children with eczema and asthma are more likely to have a mother with anxiety or depression compared with healthy children, maternal mental health may be an important mediator of the association between eczema and asthma and child mental well-being. A better understanding of the role of maternal mental health could help elucidate the mechanisms that underpin the association between eczema and asthma and child mental well-being, and if causal, could aid the development of interventions to improve the mental and physical health of children affected by these conditions.^25^

Few previous studies on the mental well-being of children with eczema or asthma have considered maternal mental health. Additional limitations to the existing literature include the frequent use of small, clinic-based, convenience samples, a lack of inclusion of potentially important confounders, and few studies focusing on younger children.^11^, ^26^, ^27^ Few studies have considered both eczema and asthma despite the frequent co-occurrence of these conditions. To address these limitations, we used data from a large, longitudinal, population-based birth cohort. We hypothesized that maternal anxiety and depression would mediate the association between rash and wheeze and child mental well-being (Figure; available at www.jpeds.com). Our first aim was to determine whether rash or wheeze symptoms were associated with internalizing (ie, anxious and depressive) or externalizing (ie, oppositional and hyperactive) behaviors at the age of 8 years, and whether duration of symptoms was an important factor. Our second aim was to determine whether these associations remained after adjustment for maternal anxiety and depression.

## Methods

Subjects were participants in the Avon Longitudinal Study of Parents and Children (ALSPAC). Details on ALSPAC have been published previously,^28^ and a fully searchable data dictionary is available online (www.bristol.ac.uk/alspac/). In brief, ALSPAC recruited pregnant women with expected dates of delivery between April 1, 1991 and December 31, 1992 who lived in a defined geographic area (Avon, United Kingdom). The children have been studied throughout their lives using maternal or self-report questionnaires and, from the age of 7 years, approximately annual research clinic visits. There were 14 062 live births, 13 988 children were alive at 1 year and over 7000 mothers completed the 8-year questionnaire. The study sample in this paper comprises the 7250 singletons with outcome data at 8 years of age. Ethical approval for the study was obtained from the ALSPAC Ethics and Law Committee and the Local Research Ethics Committees.

### Measures

#### Exposure - Child Rash, Wheeze, and Atopy

Child rash and wheeze symptoms were reported in mailed, self-completion questionnaires sent to the mother when her child was aged 6 months, 18 months, 2 years 6 months, 3 years 6 months, 4 years 9 months, 5 years 9 months, 6 years 9 months, and 7 years 7 months (Table I; available at www.jpeds.com). When questionnaires contained more than 1 question about wheeze or rash, a symptom was coded as being present if the mother reported yes to any 1 of the questions. The time periods covered by the questionnaires were categorized as infancy (up to 18 months), preschool (18 months up to 4 years 9 months), and school age (4 years 9 months up to 7 years 7 months). For each time period, symptoms were coded as being present if the mother reported that the child had the symptom in at least 1 questionnaire. For rash and wheeze separately, symptoms were categorized as ‘none’ (no symptoms in any time period); ‘early onset, transient’ (symptoms in infancy and/or preschool only); ‘persistent’ (symptoms in infancy and/or preschool, and at school age); and ‘late onset’ (symptoms at school age only). Atopy status was known for 5004 children in our sample who attended an ALSPAC clinic when aged 7.5 years; atopy was determined by skin prick testing and defined as a positive response (≥2 mm weal) to any one of *Dermatophagoides pteronyssinus*, grass, or cat allergen with a negative response to diluent solution. As previously reported, this definition identified >95% of subjects with any positive response to a wider panel of allergens.^29^

#### Outcome—Child Mental Well-Being

Child mental well-being at age 8 years 1 month was maternally reported using the parental version of the Goodman Strengths and Difficulties Questionnaire, a validated behavioral screening tool for children and adolescents.^30^ The peer and emotion subscales can be summed to give an internalizing problems score (range 0-20), and the hyperactivity and conduct subscales summed to give an externalizing problems score (range 0-20).^31^ All 4 subscales can be summed to give a total difficulties score (TDS) (range of 0-40); a higher score reflects more difficulties. For each of the TDS, externalizing and internalizing scores, children were classified as having a high score or not (high defined as >90th percentile). The cut-offs for a high score were ≥7 for internalizing, ≥10 for externalizing, and ≥15 for TDS.

#### Other Variables

Maternal anxiety and depression were measured in pregnancy and at the outcome time-point (when child was 8 years old). Depression was measured by the Edinburgh Postnatal Depression Scale. Although this measure was originally designed for use with postnatal women, none of the 10 items is specific to this period and it has been validated for use at other times^32^; it was chosen as it does not contain somatic items that could confound normal symptoms in pregnancy with depression. Anxiety was measured in pregnancy by the 8 items of the anxiety subscale of the Crown-Crisp Experiential Index^33^ and 8 years later, by the 20-trait anxiety items of the State-Trait Anxiety Inventory.^34^ Quartiles of depression and anxiety scores were calculated for use in analyses as they were not normally distributed.

A number of other child, mother, and socioeconomic variables thought to be potential confounders or mediators (based on previous literature or on theoretical grounds) were also included in analyses. Child variables were maternally reported: sex; ethnicity (White, non-White [no further disaggregation was possible due to small numbers]); age at outcome; and child wakes at night (no, once, twice or more). Socioeconomic position (SEP) was reported during pregnancy: highest maternal education (university degree, A level, O level, vocational/none); housing tenure (owned/mortgaged, privately rented, council rented, other); and financial difficulties (quartiles of score with range 0-40, where 0 is no financial difficulties). Maternal smoking (no, yes) was reported during pregnancy and at the outcome time point, and maternal insufficient sleep (no, yes) when the child was aged 7 years.

### Missing Data

Multiple imputation using chained equations was used to replace missing exposure and confounder data with predictions based on information observed in the sample. The percentage of missing data was below 10% for most variables (Table II; available at www.jpeds.com); 47% of the children had complete data, with another 40% having between 1 and 4 missing variables. Twenty imputed datasets were created and analyzed using mi estimate commands in Stata 13 (StataCorp, College Station, Texas). Complete case analysis was also performed; results were consistent with those from the imputed data and are available from authors on request. Atopy data were not imputed; a separate complete case analysis was performed for this exposure.

### Statistical Analyses

χ^2^ tests were used to assess whether distributions of the outcome and confounder variables differed by rash or wheeze status. To determine whether maternal mental health could be a mediator in the relationship between child rash or wheeze and child mental well-being, we first used multivariable logistic regression models to test for an association between the exposure and the potential mediator (child rash/wheeze and maternal anxiety/depression) and between the potential mediator and the outcome (maternal anxiety/depression and child mental well-being).^35^ Multivariable logistic regression models were then used to model the association between child rash and wheeze and the child mental well-being outcomes. Eight multivariable logistic regression models were fitted for each binary outcome (high TDS, high internalizing problems, high externalizing problems). All models adjusted for baseline confounders (child demographics, SEP, mother's age at delivery). Model 1 added rash and wheeze individually to these baseline confounders; model 2 then included both rash and wheeze together. Models 3 and 4 added maternal anxiety and depression, in pregnancy and at the outcome time point, respectively, to the variables in model 2. Model 5 adjusted for maternal anxiety and depression at both time points. Finally, we included maternal smoking (model 6), maternal sleep (model 7), and child sleep (model 8). Interaction terms were fitted to test whether the relationship between rash/wheeze and the child mental well-being outcomes differed by child sex; these were not significant and so models were adjusted for sex but not stratified. We also tested for interactions between rash and wheeze, and between maternal mental health and rash and wheeze; none of these were significant and so are not included in the models presented.

## Results

The 7250 children (3681 boys and 3569 girls) had a mean age of 8.2 years at outcome assessment. Compared with the excluded sample (children with no outcome data), study children were more likely to be of higher SEP and to have ever had a rash. The mothers of the study children reported lower anxiety and depression than the mothers of the excluded sample and were less likely to smoke (Table III; available at www.jpeds.com).

In the study sample, more mothers reported that their child had ever had a rash (71%) than wheeze (46%). The patterning of symptoms by maternal SEP differed for rash and wheeze (Table IV). Mothers whose child had wheeze were more likely to have reported financial difficulties and were less likely to have owned their own home. In contrast, mothers whose child had early onset rash were the most likely to be educated to degree level. Children with a persistent rash were the most likely to have had wheeze, and children with wheeze at school age were the most likely to have had a rash. Children with wheeze were more likely to have a mother who smoked, but there was no association between maternal smoking and child rash status (Table IV). Children with wheeze and rash at school age were the most likely to wake during the night (Table IV). Maternal sleep was correlated with child sleep; of mothers who reported insufficient sleep, 21% said their child woke at least once at night compared with 14% of mothers who reported sufficient sleep. Maternal anxiety and depression scores, in pregnancy and at the outcome time point, were associated with child rash and wheeze and with child externalizing, internalizing, and TDS (Tables IV and V and Figure; Table V available at www.jpeds.com).

**Table IV:** Summary of child mental well-being outcomes and confounders/mediators, overall and by rash and wheeze status

	Child's age when questionnaire posted to mother	Overall (n = 7250, 100%)	Rash‡				Wheeze‡			
			None	Early onset transient	Persistent	Late onset	None	Early onset transient	Persistent	Late onset
Outcome - child mental well-being (SDQ)										
TDS (% high)∗^,^†	8 years 1 month	10.5	8.4	10.1	12.2	12.7	8.8	11.2	15.4	10.9
Internalizing problems score (% high)∗^,^†	8 years 1 month	11.1	9.4	10.5	12.7	12.9	9.4	11.8	15.7	12.2
Externalizing problems score (% high)∗^,^†	8 years 1 month	10.2	7.9	10.0	12.0	11.6	8.6	10.6	16.1	8.7
Exposures - child rash and wheeze status										
Wheeze∗										
None (%)	birth to 7 years 7 months	53.6	62.9	53.1	45.3	56.5				
Early onset transient (%)		28.0	25.8	31.0	27.0	28.2				
Persistent (%)		14.1	7.8	12.3	22.1	9.8				
Late onset (%)		4.4	3.5	3.5	5.6	5.5				
Rash†										
None (%)	birth to 7 years 7 months	29.0					34.1	26.8	16.1	23.5
Early onset transient (%)		29.9					29.7	33.2	26.2	24.1
Persistent (%)		33.4					28.2	32.2	52.4	42.8
Late onset (%)		7.6					8.0	7.7	5.3	9.6
Other variables										
Maternal age at delivery (mean)∗^,^†		29.1	28.9	28.9	29.2	29.6	29.2	28.9	28.7	29.2
Socioeconomic status										
Maternal education (% with university degree)∗	Pregnancy	16.4	12.9	17.4	19.0	14.3	17.0	15.4	14.7	19.5
Housing tenure (% with mortgage/owned)†	Pregnancy	82.0	81.3	82.4	82.7	80.1	84.9	80.0	75.6	78.9
Financial difficulties (% none)†	Pregnancy	40.3	40.7	40.5	40.0	39.5	44.3	35.5	35.3	37.5
Mental health in pregnancy										
Depression Score (EPDS) (% Q4 - high)∗^,^†	Pregnancy	24.3	23.1	24.7	25.9	20.3	20.6	27.7	31.2	25.6
Anxiety score (% Q4 - high)∗^,^†	Pregnancy	19.1	18.2	18.5	20.8	17.9	15.9	22.6	24.5	19.1
Mental health at outcome time point										
Depression score (EPDS) (% Q4 - high)∗^,^†	8 years 1 month	23.5	20.3	23.1	25.8	27.6	21.8	24.7	27.3	24.3
Anxiety score (% Q4 - high)∗^,^†	8 years 1 month	24.3	21.9	24.1	25.7	28.4	23.1	25.2	27.0	25.2
Maternal smoking										
Yes in pregnancy (%)†	Pregnancy	20.3	20.4	21.8	18.8	21.2	17.2	23.7	25.0	21.9
Yes at outcome time point (%)†	8 years 1 month	19.4	19.1	21.2	18.1	19.2	16.5	22.9	22.9	21.5
Maternal sleep										
Insufficient sleep (%)∗^,^†	7 years 1 month	38.0	34.0	38.8	41.0	37.4	34.7	40.3	45.5	40.8
Child sleep										
Wakes at night (% ≥1 times per night)∗^,^†	6 years 9 months	16.8	15.0	16.0	18.0	20.9	15.2	16.4	22.1	21.3

*EPDS*, Edinburgh Postnatal Depression Scale; *SDQ*, Strengths and Difficulties Questionnaire.

∗ Difference between rash categories in distribution of variable statistically significant (*P* < .05).

† Difference between wheeze categories in distribution of variable statistically significant (*P* < .05).

‡ Numbers in each rash and wheeze category not shown as they differ across imputed datasets.

Internalizing and externalizing continuous scores were moderately correlated (r = 0.39). However, most of the children with a high score on 1 measure did not have a high score on the other measure; 482 children had a high externalizing score only, 548 children had a high internalizing score only, and 257 had both a high externalizing and internalizing score.

One-fifth of the children (20.4%) who had a skin prick test had a positive reaction, indicating atopy. Children with early onset transient rash (OR 1.61, 95% CI, 1.29-2.01) and persistent rash (3.31, 2.70-4.05) were more likely to be atopic than children who had never had a rash; there was no difference for late onset rash. For wheeze, those with persistent (4.49, 3.74-5.40) and late onset (3.24, 2.38-4.41) wheeze were more likely to be atopic than those with never wheeze, but there was no difference for early onset transient wheeze.

Children with rash had elevated odds of having a high internalizing, externalizing, and TDS relative to those who had never had a rash. ORs were higher for externalizing problems than internalizing problems, and were higher for those with a rash at school age (whether persistent or late onset) compared with those with early onset transient symptoms (Table VI, model 1). Children with persistent wheeze, and to a lesser extent early onset transient wheeze, had higher odds of internalizing and externalizing problems, and of having a high TDS, than children who had never wheezed. There was no association between late onset wheeze and any of the mental well-being outcomes. Rash and wheeze were associated with the outcomes independent of each other, although including both in the same model did attenuate associations for persistent rash and persistent wheeze for all outcomes (Table VI, model 2).

**Table VI:** Association between child rash and wheeze status and child mental well-being at age 8 years

	OR (95% CI)							
	Model 1 Rash and wheeze in separate models	Model 2 Rash and wheeze in same model	Model 3 M2 + maternal anxiety and depression in pregnancy	Model 4 M2 + maternal anxiety and depression when child 8 years	Model 5 M2 + maternal anxiety and depression at both time points	Model 6 M5 + maternal smoking	Model 7 M5 + maternal sleep	Model 8 M5 + child sleep
High internalizing symptoms								
Rash (reference = no rash)								
Early onset transient	1.14 (0.92-1.42)	1.10 (0.89-1.38)	1.09 (0.88-1.36)	1.04 (0.83-1.31)	1.04 (0.83-1.31)	1.05 (0.83-1.31)	1.03 (0.82-1.30)	1.04 (0.83-1.30)
Persistent	1.42 (1.16-1.74)†	1.31 (1.07-1.61)†	1.28 (1.04-1.57)∗	1.21 (0.98-1.50)	1.21 (0.98-1.50)	1.21 (0.98-1.50)	1.20 (0.97-1.48)	1.20 (0.97-1.49)
Late onset	1.46 (1.07-1.99)∗	1.43 (1.05-1.95)∗	1.44 (1.06-1.98)∗	1.28 (0.93-1.77)	1.30 (0.94-1.79)	1.30 (0.94-1.79)	1.30 (0.94-1.80)	1.27 (0.92-1.75)
Wheeze (reference = no wheeze)								
Early onset transient	1.22 (1.02-1.46)∗	1.21 (1.01-1.44)∗	1.15 (0.96-1.38)	1.17 (0.97-1.40)	1.15 (0.96-1.38)	1.15 (0.96-1.38)	1.14 (0.95-1.37)	1.15 (0.95-1.38)
Persistent	1.67 (1.35-2.06)‡	1.58 (1.28-1.96)‡	1.48 (1.19-1.83)‡	1.54 (1.23-1.92)‡	1.50 (1.20-1.87)^‡^	1.50 (1.20-1.87)‡	1.47 (1.18-1.84)†	1.45 (1.16-1.81)†
Late onset	1.30 (0.89-1.91)	1.25 (0.85-1.84)	1.20 (0.81-1.76)	1.21 (0.82-1.78)	1.20 (0.81-1.77)	1.20 (0.81-1.78)	1.18 (0.80-1.75)	1.16 (0.79-1.72)
High Externalizing Symptoms								
Rash (reference = no rash)								
Early onset transient	1.33 (1.06-1.67)∗	1.29 (1.03-1.63)∗	1.28 (1.02-1.62)∗	1.22 (0.97-1.54)	1.22 (0.97-1.54)	1.21 (0.96-1.53)	1.21 (0.96-1.53)	1.21 (0.96-1.52)
Persistent	1.74 (1.40-2.15)‡	1.61 (1.29-2.00)‡	1.58 (1.27-1.97)‡	1.47 (1.17-1.83)†	1.47 (1.18-1.84)†	1.48 (1.18-1.85)†	1.46 (1.17-1.83)†	1.46 (1.17-1.84)†
Late onset	1.62 (1.17-2.25)†	1.60 (1.15-2.22)†	1.62 (1.17-2.25)†	1.43 (1.03-2.01)∗	1.45 (1.04-2.03)∗	1.44 (1.03-2.02)∗	1.45 (1.04-2.03)∗	1.41 (1.00-1.98)∗
Wheeze (reference = no wheeze)								
Early onset transient	1.13 (0.93-1.37)	1.10 (0.91-1.34)	1.07 (0.88-1.30)	1.06 (0.87-1.29)	1.05 (0.86-1.29)	1.04 (0.85-1.27)	1.05 (0.86-1.28)	1.05 (0.86-1.28)
Persistent	1.74 (1.41-2.15)‡	1.59 (1.28-1.97)‡	1.52 (1.22-1.89)‡	1.55 (1.25-1.94)‡	1.54 (1.24-1.93)^‡^	1.53 (1.23-1.91)‡	1.52 (1.22-1.90)‡	1.47 (1.18-1.84)†
Late onset	0.99 (0.62-1.57)	0.93 (0.59-1.47)	0.90 (0.57-1.44)	0.89 (0.56-1.41)	0.88 (0.56-1.41)	0.87 (0.55-1.39)	0.88 (0.55-1.40)	0.85 (0.54-1.36)
High TDS								
Rash (reference = no rash)								
Early onset transient	1.25 (0.99-1.59)	1.21 (0.96-1.54)	1.20 (0.95-1.52)	1.14 (0.90-1.45)	1.15 (0.90-1.46)	1.15 (0.90-1.45)	1.13 (0.89-1.44)	1.13 (0.89-1.44)
Persistent	1.61 (1.31-1.98)‡	1.49 (1.21-1.84)‡	1.46 (1.18-1.81)‡	1.36 (1.09-1.68)†	1.36 (1.10-1.69)†	1.37 (1.10-1.70)†	1.35 (1.08-1.67)†	1.35 (1.09-1.68)†
Late onset	1.68 (1.23-2.29)†	1.65 (1.21-2.25)†	1.69 (1.23-2.31)†	1.47 (1.06-2.03)∗	1.50 (1.08-2.07)∗	1.50 (1.08-2.07)∗	1.50 (1.09-2.08)∗	1.45 (1.04-2.01)∗
Wheeze (reference = no wheeze)								
Early onset transient	1.20 (1.00-1.44)	1.17 (0.98-1.41)	1.12 (0.93-1.35)	1.13 (0.93-1.37)	1.11 (0.92-1.35)	1.11 (0.91-1.35)	1.11 (0.91-1.34)	1.11 (0.91-1.35)
Persistent	1.66 (1.34-2.04)‡	1.54 (1.24-1.91)‡	1.43 (1.15-1.78)‡	1.49 (1.20-1.87)‡	1.46 (1.17-1.83)†	1.46 (1.16-1.82)†	1.42 (1.14-1.78)†	1.38 (1.10-1.73)†
Late onset	1.24 (0.81-1.90)	1.18 (0.77-1.80)	1.13 (0.73-1.73)	1.13 (0.73-1.74)	1.12 (0.72-1.73)	1.11 (0.72-1.72)	1.10 (0.71-1.70)	1.07 (0.69-1.66)

All models adjust for child sex, age, ethnicity, mother's age, maternal education, financial difficulties, housing tenure.

Model 1: separate model for rash and wheeze status.

Model 2: rash status, wheeze status.

Model 3: Model 2 plus maternal anxiety and depression in pregnancy.

Model 4: Model 2 plus maternal anxiety and depression when child aged 8 years.

Model 5: Model 2 plus maternal anxiety and depression in pregnancy and when child aged 8 years.

Model 6: Model 5 plus maternal smoking in pregnancy and when child aged 8 years.

Model 7: Model 5 plus maternal sleep.

Model 8: Model 5 plus child sleep.

∗ *P* < .05.

† *P* < .01.

‡ *P* < .001.

Maternal anxiety and depression when the child was 8 years old, but not during pregnancy, accounted for the association between child rash and internalizing symptoms and partly for the association with externalizing symptoms (Table VI, models 3 and 4). On the other hand, maternal anxiety and depression during pregnancy, rather than in childhood, appeared more relevant in accounting for the association between internalizing and externalizing symptoms and child persistent wheeze. Further adjustment for maternal smoking or maternal sleep did not modify these associations (Table VI, model 6 and 7). Child sleep explained some of the association between the outcomes and persistent wheeze, but not rash (Table VI, model 8).

In the subsample of children who had their atopy status determined by skin prick test (n = 5004), there was no association between atopy and the odds of having a high internalizing (OR 0.91, 95% CI, 0.72-1.15), externalizing (0.91, 0.72-1.16), or total difficulties (1.03, 0.82-1.30) score in age-adjusted models. Complete confounder data was available for 3584 of these children; adjustment made little difference to the associations observed (Table VII; available at www.jpeds.com).

## Discussion

Both rash and wheeze status were related to child mental well-being at the age of 8 years in our large, population-based cohort study; persistent and late onset rash, and persistent wheeze, were associated with both internalizing and externalizing symptoms. These results confirm, in a large prospective study, previous results of increased internalizing^5^, ^10^, ^14^, ^26^, ^27^ and externalizing^11^, ^36^ problems in children with eczema and asthma. We have demonstrated the importance of including measures of maternal anxiety and depression, both during and after pregnancy, when examining the relationship between rash and wheeze and child mental well-being. For persistent and late onset rash, we found that maternal anxiety and depression, particularly after the birth of the child rather than during pregnancy, accounted for the association with internalizing symptoms and partly for externalizing symptoms. Worse sleep did not mediate the association. However, maternal anxiety and depression at both time points attenuated the association with persistent wheeze. Worse child sleep also contributed to the association with persistent wheeze.

In our study, children who only had symptoms in infancy or preschool tended to have poorer mental well-being at 8 years compared with those with no symptoms at any age; early onset transient wheeze was associated with more internalizing problems, and early onset transient rash with more externalizing problems. Similarly, a large German cohort study found that children who cleared their eczema by age 2 years tended to have more emotional and conduct problems at age 10 years than children who had never had eczema.^14^ We found generally similar sized associations for persistent and late onset rash, which contrasts with the German study which found that emotional problems were greater the longer eczema persisted^14^; age at outcome and categories of symptom duration differed between the studies, which may explain the different findings. We mutually adjusted for rash and wheeze status to account for the fact that these conditions often coexist, and found both symptoms to be independently associated with the outcomes. There is limited evidence from previous literature on the relative impact of eczema and asthma on child mental well-being, and findings are mixed. In the German study, eczema was associated with mental well-being independent of atopic comorbidity but associations for asthma were not reported.^14^ In 4 other studies that included measures of both eczema and asthma, all of which had ADHD as their outcome, 3 found only eczema was independently associated with ADHD in fully adjusted models,^12^, ^13^, ^36^ but another study reported that asthma, but not eczema, was associated with ADHD.^37^

Our mediation model assumes that child rash and wheeze predict maternal anxiety and depression, which in turn co-occur with poor child mental well-being (ideally child mental well-being would have been measured after maternal anxiety and depression in order to truly establish mediation). As maternal anxiety and depression in pregnancy occur before child rash and wheeze develop, they cannot mediate the association; however, child rash and wheeze could be a mechanism through which maternal depression and anxiety in pregnancy predict later child well-being.

A true mediating mechanism could involve both behavioral and biological pathways. Caregivers with poor mental health feel less empowered to deal with their child's condition or to manage it effectively, are less knowledgeable about their child's medications, and their children are at increased risk of poor medication adherence, increased hospitalization, and lower asthma-related quality of life.^38^, ^39^, ^40^, ^41^, ^42^ Therefore, poor mental health may impair a mother's ability to cope with the demands of her child's condition, including administering the appropriate treatment regimes, resulting in a worsening of her child's physical symptoms.^43^

Many children with asthma and eczema, and consequently their parents, suffer from disrupted sleep,^21^, ^44^, ^45^, ^46^ and previous studies have reported that sleep quality is a key factor in the association between eczema, asthma, and behavioral problems in children.^13^, ^44^, ^47^, ^48^ In the present study, maternal and child sleep were related, but only child sleep attenuated the association between wheeze and internalizing and externalizing problems. Maternal mental health may, therefore, have direct effects on a child's mental health, as well as indirect effects via poor disease management resulting in a worsening of symptoms, which in turn leads to disrupted sleep and an increase in behavioral difficulties. However, such pathways are likely to be cyclical as poor mental well-being in asthmatic children is associated with increased disease severity over time.^49^ Furthermore, a child's physical symptoms may affect how the mother parents her child; studies have found perceived child vulnerability^50^ and negative parenting behaviors^51^ to mediate the association between parental depression and child internalizing symptoms in children with asthma. This highlights the complexity of the relationships between child physical symptoms and child and maternal mental health.

In addition to the potential behavioral pathways described above, there may be biological pathways linking eczema and asthma to mental well-being. Eczema and asthma in children are both associated with stress, which may be mediated by common underlying immunologic mechanisms, such as atopy and systemic inflammatory responses.^11^ It is thought that systemic inflammatory responses and stress could influence behavior and emotions via effects on the brain.^11^, ^52^ Maternal mental health could play a role in such a biological pathway, as parental stress and depression have been reported to predict increases in a child's inflammatory profile.^41^ There was no association between atopy and child mental well-being in our study, suggesting that associations between rash and wheeze and mental well-being were not mediated by allergy; however, we did not have relevant biomarkers of systemic inflammation to explore this pathway further.

Rather than a mediating mechanism, it is possible in this observational analysis that the attenuating effect of maternal mental health is due to confounding. For example, lower SEP is associated with both maternal mental health and more child wheeze. However, we found that the role of maternal mental health remained after adjustment for three measures of SEP. Furthermore, confounding by SEP is not likely given the association between maternal SEP and child rash is in the opposite direction. Mothers with poorer mental health are more likely to smoke, and maternal smoking is associated with both child asthma and eczema, and child mental health.^53^ However, in this study maternal smoking did not explain any of the excess risk of behavioral problems associated with child rash or wheeze.

A further potential explanation is reporting bias. A mother's mental health may influence how she perceives and reports both her child's symptoms and behavior.^49^ This is important as the majority of studies, including ours, use maternal reports. All of our measures, except for atopy, were maternally reported thereby raising the possibility of bias from shared method variance. Finally, associations could be due to reverse causality as maternal mental health may be reduced as a result of the child having poor mental health. We have not been able to test for reverse causality in our study, and it is difficult to disentangle these potential mechanisms in observational studies.

If maternal anxiety and depression have a true mediating role, treatment to improve maternal mental health could optimize the well-being of children with asthma and eczema. The idea of ‘treating the parents to heal the child’ has been proposed for children with asthma and eczema, but a better understanding of the role of parental psychological well-being in child health is needed.^25^ Although there is some limited evidence that interventions, such as cognitive behavioral therapy for anxiety disorders, can improve asthma-related quality of life in both adults^54^ and children,^55^ we are unaware of any intervention to date that has examined the impact of improving maternal anxiety and depression on the well-being of children with asthma or eczema.

Strengths of this current study include the use of a large, longitudinal, population-based cohort, and the inclusion of several potentially important confounders. In addition, we included both rash and wheeze, and demonstrated the importance of accounting for the duration of symptoms in the association of rash and wheeze with later mental health. Our study also has limitations. We had no objective measure of rash and wheeze severity, and not all reported rash and wheeze will be due to eczema or asthma, which may limit comparison with previous studies. However, the wheeze questions are consistent with those used by the International Study of Asthma and Allergies in Childhood and are a reliable way of diagnosing asthma in large epidemiologic studies where direct physician assessment would not be feasible.^56^ Also, a subset of the children had their skin assessed at an ALSPAC clinic when aged 49 months; observed eczema was associated with maternally reported child rash. It is possible that some children developed symptoms in the 6-month period between the last measure of rash/wheeze status (at age 7 years 7 months) and the outcome measures (at age 8 years) and so may have had their symptom status misclassified; however, such errors would lead to our estimates being conservative. Child sleep quality was measured at age 6 years 9 months and maternal sleep at age 7 years 1 month; these variables may not reflect sleep quality at the outcome time point, particularly if rash or wheeze symptoms changed after sleep quality was measured. Lastly, in this study we have only considered child mental well-being at age 8 years; we have not considered how mental well-being, or its associations with rash and wheeze, develop over time.

In conclusion, maternal anxiety and depression explained some of the association between child rash and wheeze and externalizing and internalizing symptoms in childhood. Further research would help determine whether this is because maternal mental health mediates the association, or whether it is due to reporting bias, confounding or reverse causality. A better understanding of the role of maternal mental health could help in the development of interventions to improve the quality of life of affected children and their families.

## Figures and Tables

**Figure:**
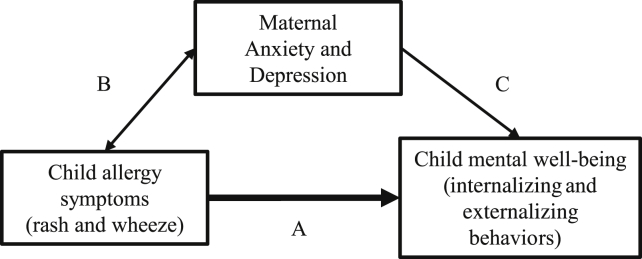
Theoretical model of role of maternal anxiety and depression in association between child allergic symptoms and child mental well-being.

**Table I:** ALSPAC questionnaire items on rash and wheeze

Child's age when questionnaire sent to mother	“Has your child had….” (since birth for 6-month questionnaire, since the previous questionnaire time point for all others)
Rash	
6 months, 18 months	1. A rash in the joints and creases of his/her body (eg, behind the knees, under the arms)?
	2. An itchy, dry, oozing or crusted rash on the face, forearms or shins?
2 years 6 months, 3 years 6 months, 4 year 9 months, 5 years 9 months, 6 years 9 months	1. An itchy, dry skin rash in the joints and creases of his/her body (eg, behind the knees, elbows, under the arms)?
	2. An itchy, dry rash on his hands?
	3. An itchy, dry rash on his feet?
7 years 7 months	1. A rash?
Wheeze	
6 months, 18 months, 2 years 6 months, 3 years 6 months, 4 years 9 months, 5 years 9 months, 6 years 9 months	1. Wheezing with whistling on his chest when he/she breathed?
	2. Wheezing?
7 years 7 months	1. Wheezing?

**Table II:** Description of missing data for each of the exposure and confounder variables

	n (%) missing (7250 = 100%)∗
Child sex	0
Mother's age at delivery	0
Child ethnic group	295 (4.1)
Housing tenure	186 (2.6)
Maternal education	184 (2.5)
Financial difficulties	363 (5.0)
Anxiety in pregnancy	719 (9.9)
Depression in pregnancy	624 (8.6)
Anxiety when child aged 8 years	536 (7.4)
Depression when child aged 8 years	139 (1.9)
Maternal smoking in pregnancy	63 (0.87)
Maternal smoking when child 8 years	27 (0.37)
Maternal sleep	701 (9.7)
Child sleep	800 (11.0)
Wheeze	
6 months	323 (4.5)
18 months	310 (4.3)
2 years 6 months	490 (6.8)
3 years 6 months	484 (6.7)
4 years 9 months	495 (6.8)
5 years 9 months	702 (9.7)
6 years 9 months	740 (10.2)
7 years 7 months	599 (8.3)
Rash	
6 months	374 (5.2)
18 months	321 (4.4)
2 years 6 months	547 (7.5)
3 years 6 months	480 (6.6)
4 years 9 months	886 (12.2)
5 years 9 months	1126 (15.5)
6 years 9 months	980 (13.5)
7 years 7 months	610 (8.4)

∗ The study sample was defined as singleton children with complete outcome data, n = 7250.

**Table III:** Comparison of study population and those with incomplete data

	Sample∗ (n = 7250)		Complete case† (n = 5513)	Excluded sample‡ (N = 6367)
	Imputed	Unimputed		
		N = 7250		
Child high internalizing problems score				
High (%)	11.1	11.1	10.6	/
		N = 7250		
Child high externalizing problems score				
High (%)	10.2	10.2	9.6	/
		N = 7250		
Child high TDS				
High (%)	10.5	10.5	9.7	/
		N = 6934		N = 2246
Child rash§				
None (%)	29.0	30.5	30.8	37.4
Early onset transient (%)	29.9	30.9	30.6	34.7
Early onset persistent (%)	33.4	31.3	31.2	22.8
Late onset (%)	7.6	7.3	7.4	5.1
		N = 6958		N = 2296
Child wheeze§				
None (%)	53.6	54.8	55.4	53.5
Early onset transient (%)	28.0	27.6	27.2	30.5
Early onset persistent (%)	14.1	13.5	13.3	13.0
Late onset (%)	4.4	4.1	4.1	3.0
		N = 7250		N = 6367
Child sex				
Female (%)	49.2	49.2	48.9	47.5
		N = 6955		N = 4814
Child ethnic group				
White (%)	96.3	96.4	96.9	92.9
		N = 7250		N = 6367
Mother's age at delivery				
Mean (years)	29.1	29.1	29.3	26.7
		N = 7064		N = 5639
Housing tenure				
Owned/mortgaged (%)	82.0	82.3	85.2	62.0
Privately rented (%)	5.7	5.6	4.8	9.3
Council rented (%)	9.4	9.2	7.8	24.4
Other (%)	3.0	2.9	2.2	4.3
		N = 7066		N = 5036
Maternal education				
Degree (%)	16.4	16.6	17.1	7.6
A-level (%)	26.1	26.3	27.3	17.3
O-Level (%)	35.3	35.2	35.7	33.7
None/Vocational (%)	22.3	22.0	19.8	41.3
		N = 6531		N = 4935
Anxiety in pregnancy				
Lowest quartile (%)	31.6	31.8	32.8	28.3
Highest quartile (%)	19.1	18.9	18.2	24.8
		N = 6626		N = 5034
Depression in pregnancy				
Lowest quartile (%)	22.0	22.2	22.8	17.2
Highest quartile (%)	24.3	23.9	22.9	33.0
		N = 6714		N = 212
Anxiety when child aged 8 years				
Lowest quartile (%)	25.3	25.6	26.1	25.9
Highest quartile (%)	24.3	23.9	23.0	28.3
		N = 7111		N = 253
Depression when child aged 8 years				
Lowest quartile (%)	30.0	30.0	31.0	29.6
Highest quartile (%)	23.5	23.5	21.9	27.3
		N = 7187		N = 5861
Maternal smoking in pregnancy				
Yes (%)	20.3	20.2	18.7	35.2
		N = 7223	N = 5498	N = 294
Maternal smoking when child 8 years				
Yes (%)	19.4	19.4	18.1	22.8
		N = 6549	N = 5165	N = 1514
Maternal sleep				
Insufficient sleep (%)	38.0	38.0	37.7	39.8
		N = 6450	N = 5121	N = 1639
Child wakes at night				
Never (%)	83.2	83.6	84.3	82.3
Once (%)	14.3	14.2	13.8	14.0
Twice or more (%)	2.4	2.2	1.8	3.8

∗ The study sample was defined as singleton children with complete outcome data, n = 7250. Missing covariates were imputed. Table compares imputed and unimputed variables for the study sample. For the unimputed data, denominators differ by variable and are stated in the Table. The imputed sample was used in analysis.

† The complete case population consists of singleton children who have complete outcome, exposure, and key confounder data. This sample was used in a sensitivity analysis.

‡ The 6367 children in the excluded sample are singletons alive at 1 year who do not have outcome data; denominators differ by variable and are stated in the Table.

§ For the unimputed, complete case, and excluded samples, the overall rash and wheeze variables were derived for children who had rash/wheeze reported at least once in each time period.

**Table V:** Association between maternal depression and anxiety and child mental well-being outcomes (high internalizing, externalizing, and total difficulties)

	OR (95% CI)∗		
	High internalizing	High externalizing	High TDS
Pregnancy			
Maternal depression			
Low (Q1, reference)			
Q2	1.28 (0.97-1.68)	1.10 (0.84-1.44)	1.03 (0.76-1.38)
Q3	1.73 (1.35-2.21)†	1.41 (1.09-1.83)†	1.66 (1.28-2.15)†
High (Q4)	2.58 (2.01-3.32)†	2.05 (1.59-2.64)†	2.69 (2.07-3.48)†
Maternal anxiety			
Low (Q1, reference)			
Q2	1.42 (1.11-1.81)†	1.03 (0.80-1.32)	1.19 (0.92-1.54)
Q3	1.92 (1.54-2.41)†	1.43 (1.14-1.79)†	1.87 (1.48-2.35)†
High (Q4)	2.75 (2.19-3.45)†	1.96 (1.56-2.46)†	2.65 (2.11-3.34)†
Outcome time point			
Maternal depression			
Low (Q1, reference)			
Q2	1.53 (1.19-1.97)†	1.76 (1.36-2.28)†	1.85 (1.41-2.43)†
Q3	2.27 (1.78-2.89)†	2.18 (1.69-2.82)†	2.73 (2.10-3.55)†
High (Q4)	4.03 (3.23-5.04)†	4.04 (3.20-5.10)†	5.24 (4.11-6.67)†
Maternal anxiety			
Low (Q1, reference)			
Q2	1.42 (1.11-1.81)†	1.03 (0.80-1.32)	1.19 (0.92-1.54)
Q3	1.92 (1.54-2.41)†	1.43 (1.14-1.79)†	1.87 (1.48-2.35)†
High (Q4)	2.75 (2.19-3.45)†	1.96 (1.56-2.46)†	2.65 (2.11-3.34)†

∗ Adjusted for maternal age at birth, financial difficulties, maternal education, housing tenure.

† *P* < .05.

**Table VII:** Association between child atopy status and child mental well-being at age 8 years

	OR (95% CI)							
	Model 1 + age	Model 2 M1 + SEP	Model 3 M2 + maternal anxiety and depression in pregnancy	Model 4 M2 + maternal anxiety and depression when child 8 years	Model 5 M2 + maternal anxiety and depression at both time points	Model 6 M5 + maternal smoking	Model 7 M5 + maternal sleep	Model 8 M5 + child sleep
Atopy (reference = no atopy)								
Complete case	N = 5004	N = 4619	N = 4295	N = 4322	N = 4029	N = 4018	N = 3780	N = 3709
High Internalizing score	0.91 (0.72-1.15)	0.89 (0.69-1.13)	0.92 (0.71-1.19)	0.88 (0.68-1.14)	0.89 (0.68-1.17)	0.89 (0.68-1.17)	0.84 (0.63-1.12)	0.81 (0.60-1.08)
High Externalizing score	0.91 (0.72-1.16)	0.95 (0.74-1.22)	0.93 (0.72-1.21)	0.88 (0.68-1.15)	0.88 (0.67-1.16)	0.89 (0.67-1.17)	0.86 (0.65-1.14)	0.86 (0.64-1.15)
High TDS	1.03 (0.82-1.30)	1.04 (0.81-1.33)	1.07 (0.83-1.38)	0.99 (0.76-1.28)	1.02 (0.78-1.34)	1.02 (0.78-1.35)	0.97 (0.73-1.29)	0.98 (0.74-1.32)
All models restricted to those with complete data	N = 3584	N = 3584	N = 3584	N = 3584	N = 3584	N = 3584	N = 3584	N = 3584
High internalizing score	0.85 (0.64-1.13)	0.84 (0.63-1.12)	0.84 (0.62-1.12)	0.81 (0.60-1.09)	0.81 (0.60-1.09)	0.81 (0.61-1.10)	0.81 (0.60-1.09)	0.80 (0.59-1.08)
High externalizing score	0.85 (0.63-1.13)	0.85 (0.64-1.14)	0.85 (0.64-1.14)	0.85 (0.64-1.14)	0.83 (0.62-1.12)	0.84 (0.62-1.13)	0.84 (0.62-1.13)	0.83 (0.62-1.12)
High TDS	0.97 (0.73-1.29)	0.98 (0.74-1.31)	0.98 (0.74-1.31)	0.96 (0.71-1.29)	0.95 (0.71-1.28)	0.96 (0.71-1.29)	0.96 (0.71-1.29)	0.95 (0.71-1.28)

Model 1: child sex, age (at skin prick test and at outcome).

Model 2: Model 1 plus ethnicity, mother's age, maternal education, financial difficulties, housing tenure.

Model 3: Model 2 plus maternal anxiety and depression in pregnancy.

Model 4: Model 2 plus maternal anxiety and depression when child aged 8 years.

Model 5: Model 2 plus maternal anxiety and depression in pregnancy and when child aged 8 years.

Model 6: Model 5 plus maternal smoking in pregnancy and when child aged 8 years.

Model 7: Model 5 plus maternal sleep.

Model 8: Model 5 plus child sleep.
